# Post-Hoc Analysis of a Multicenter Clinical Trial: Correlation of Coagulation Factor Changes and MRI-Defined Treatment Outcomes After Sclerotherapy for Venous Malformations

**DOI:** 10.3390/jcm14030905

**Published:** 2025-01-30

**Authors:** Tadashi Nomura, Mine Ozaki, Keigo Osuga, Masakazu Kurita, Ayato Hayashi, Shunsuke Yuzuriha, Noriko Aramaki-Hattori, Makoto Hikosaka, Taiki Nozaki, Michio Ozeki, Junko Ochi, Shimpei Akiyama, Yasumasa Kakei, Keiko Miyakoda, Naoko Kashiwagi, Takahiro Yasuda, Yuki Iwashina, Tsuyoshi Kaneko, Hiroto Terashi, Kiyonori Harii

**Affiliations:** 1 Department of Plastic Surgery, Kobe University Graduate School of Medicine, Kobe 655-0865, Japan; 2 Department of Plastic Surgery, Kyorin University Faculty of Medicine, Tokyo 181-8611, Japan; zakimin@nifty.com (M.O.);; 3 Department of Diagnostic Radiology, Osaka Medical and Pharmaceutical University, Osaka 569-0801, Japan; 4 Department of Plastic and Reconstructive Surgery, The University of Tokyo Hospital, Tokyo 113-8655, Japan; 5 Department of Plastic and Reconstructive Surgery, Yokohama City University, Yokohama 236-0004, Japan; 6 Department of Plastic and Reconstructive Surgery, Juntendo University School of Medicine, Tokyo 113-0033, Japan; 7 Department of Plastic and Reconstructive Surgery, Shinshu University School of Medicine, Matsumoto 390-8621, Japan; 8 Department of Plastic and Reconstructive Surgery, Keio University School of Medicine, Tokyo 160-8582, Japan; 9 Department of Plastic and Reconstructive Surgery, National Center for Child Health and Development, Tokyo 157-8535, Japan; 10 Department of Radiology, Keio University School of Medicine, Tokyo 160-8582, Japan; 11 Department of Pediatrics, Graduate School of Medicine, Gifu University, Gifu 501-1112, Japan; 12 Department of Diagnostic Radiology, Suita Tokushukai Hospital, Osaka 565-0814, Japan; 13 Department of Radiology, Kyoto Prefectural University of Medicine, Kyoto 602-8566, Japan; 14 Department of Oral and Maxillofacial Surgery, Kobe University Graduate School of Medicine, Kobe 650-0017, Japan; 15 Clinical and Translational Research Center, Kobe University Hospital, Kobe 650-0017, Japan; 16 Department of Medical Devices, Kobe University Graduate School of Medicine, Kobe 650-0017, Japan

**Keywords:** ethanolamine oleate, venous malformations, sclerotherapy, coagulation factor

## Abstract

**Background/Objectives:** The therapeutic efficacy of percutaneous sclerotherapy (PS) for venous malformations (VMs) based on volumetric magnetic resonance imaging (MRI) measurements and its association with early post-treatment coagulation markers remains unexplored. This study evaluates the therapeutic efficacy of 5% monoethanolamine oleate (EO)-based PS in treating difficult-to-resect VMs using volumetric MRI and investigates its association with early changes in coagulation markers. **Methods:** This post-hoc analysis utilized data from a prospective, open-label, multicenter clinical trial initiated on 1 January 2021. The correlation between MRI-determined volume reduction and post-sclerotherapy changes in coagulation markers was assessed. **Results:** Between January 2021 and April 2023, 44 patients underwent EO-based PS. Based on a ≥ 20% VM volume reduction, patients were classified into “achieved” (*n* = 26; 59.1%) and “non-achieved” (*n* = 18; 40.9%) groups. D-dimer levels significantly increased on postoperative day 1 (POD1) compared with pretreatment screening (*p* < 0.001), whereas fibrinogen and prothrombin international normalized ratio levels remained unchanged. In the achieved group, a significant correlation was observed between the volume reduction rate and the administered EO dose per lesion volume (mL/cm^3^; Spearman’s ρ = 0.43, *p* = 0.03). The non-achieved group showed significantly higher D-dimer elevation than the achieved group (*p* = 0.03). **Conclusions:** This is the first multicenter study to evaluate EO-based PS efficacy for VMs using volumetric MRI and explore its relationship with early post-treatment coagulation markers. Elevated D-dimer levels on POD1 were not predictive of treatment efficacy, highlighting their limited clinical utility in assessing therapeutic response.

## 1. Introduction

Venous malformation (VM) is a fetal vascular disorder associated with symptoms such as pain, functional impairment, and cosmetic disfigurement [1,2,3]. A study by the Japanese Ministry of Health, Labour and Welfare (Project on Intractable Hemangioma, Vascular Malformation, Lymphangioma, Lymphangiomatosis and Related Diseases in Fiscal 2014) estimated that approximately 20,000 individuals in Japan have VM. About half of these patients are believed to have lesions involving vital muscles or nerves, making surgical resection challenging [4,5]. For these patients, percutaneous sclerotherapy (PS) is typically the treatment of choice [2,6,7,8,9]. The 2017 Japanese guidelines recommend PS for its effectiveness in alleviating symptoms and reducing lesion size (weak recommendation, level D) [10].

While the efficacy of PS for VMs has been demonstrated, few studies have utilized accurate volumetric magnetic resonance imaging (MRI) measurements to evaluate treatment outcomes. Although changes in coagulation markers following sclerotherapy are well documented, the relationship between PS efficacy and coagulation markers remains poorly understood. Particularly, D-dimer levels usually increase after sclerotherapy. Based on clinical experience, we observe that a higher therapeutic effect of sclerotherapy is associated with a significant increase in D-dimer levels. Therefore, we hypothesized that the volume reduction effect of sclerotherapy is higher in cases where D-dimer levels increase after treatment. This study evaluates the therapeutic effects of PS for VMs using accurate volumetric MRI measurements in a multicenter clinical trial and investigates the relationship between treatment response and early post-treatment coagulation markers.

## 2. Materials and Methods

### 2.1. Study Design and Participants

This study is a post-hoc analysis of clinical data from a non-randomized, prospective, open-label, multicenter clinical trial initiated on 1 January 2021. The trial was designed to evaluate the efficacy and safety of PS using 5% monoethanolamine oleate (EO) for difficult-to-resect VMs. It was registered in the Japan Registry of Clinical Trials (identifier: jRCT2051200046) under the recommendations of the International Committee of Medical Journal Editors (registered on 25 August 2020: jRCT2051200046). The primary study categorized cystic and diffuse VMs into separate cohorts to assess lesion reduction efficacy as part of the approval strategy. For this post-hoc analysis, cystic and diffuse lesions were combined into a single cohort to examine the relationship between coagulation factor trends and treatment efficacy. This study was conducted at eight academic hospitals, with a 3-month observation period following the intervention. The study protocol adhered to the SPIRIT statement. Inclusion criteria were as follows: (i) the presence of VMs deemed difficult to resect, with sclerotherapy identified as the first-line treatment option by the principal or sub-investigator. Difficulty in resection was defined as involving a high risk of functional loss or significant cosmetic disfigurement affecting daily life; (ii) the presence of at least one VM with a major axis of ≥ 30 mm in the extremities or ≥ 20 mm in the head and neck region on MRI or computed tomography (CT); and (iii) the absence of thrombus or organized tissue impeding image evaluation or judgment of the target VM. Exclusion criteria encompassed multiple organ failure or disseminated intravascular coagulation; current or prior use of propranolol or Chinese herbal medicines; diabetes mellitus with HbA1c ≥ 8.0; autoimmune disorders, grade C liver dysfunction (Child–Pugh classification), renal dysfunction (eGFR < 60 mL/min/1.73 m^2^), or cardiac dysfunction (NYHA class II or higher); sclerotherapy within 6 months prior to consent; allergy to EO or angiographic X-ray contrast medium; recent surgery exceeding 45 min within 2 weeks prior to consent; participation in other clinical studies within 4 weeks prior to consent; pregnant or cases deemed inappropriate by the principal investigator.

A retrospective chart review of all 44 participants was conducted. Screening evaluations, including MRI, blood tests, chest X-rays, and ECGs, were performed after obtaining informed consent. Blood tests were repeated the day after EO administration to measure changes in coagulation factors, such as fibrinogen (mg/dL), D-dimer (µg/mL), and prothrombin time-international normalized ratio (PT-INR). Sclerotherapy was performed under general anesthesia within 1 month of consent, and treatment outcomes, including MRI, were evaluated 3 months after sclerotherapy.

### 2.2. MRI Imaging Methods and Volumetric Measurements

The required MRI sequences included T1-weighted, T2-weighted, T2 fat-suppressed or short tau inversion recovery (STIR), and 3D-T2 fat-suppressed images. Axial sections were mandatory for all modalities, while coronal sections were permissible for 3D imaging if time constraints precluded axial acquisition. Slice thickness was typically 3–5 mm, with 7–8 mm acceptable for larger lesions. MRI systems with a static magnetic field strength of 1.5 T or higher were recommended.

MRI images were independently evaluated by two board-certifi ed radiologists using OsiriX image analysis software version 12.5.1 (PixMeo SARL, Geneva, Switzerland), with the average of their measurements serving as the final value. Each radiologist performed two measurements blinded to the other’s measurements, and the average of the measurements was considered the evaluator’s assessment value. Finally, the mean of each evaluation value was used as the final evaluation value. The inter-observer correlation coefficient (ICC) was calculated to evaluate the reproducibility of the measurement values among the raters. The primary endpoint of the original analysis was defined as achieving ≥20% volumetric reduction of VMs at 3 months post-intervention for cystic lesions. This threshold was based on the assumption that successful treatment would lead to symptomatic improvement and either complete response (complete lesion resolution) or partial response (≥20% lesion volume reduction). Previous data on MRI-based assessments of EO-treated VMs are limited. Kaji et al. reported a median reduction in lesion volume of approximately 25% in 60 patients with VMs after undergoing sclerotherapy [7]. This suggests that over 50% of patients could achieve ≥20% volume reduction. Treatment response was stratified into two groups: the “achieved” group, comprising patients with ≥20% reduction, and the “non-achieved” group, comprising those who did not meet this criterion.

### 2.3. Sclerotherapy Technique

**Figure 1:**
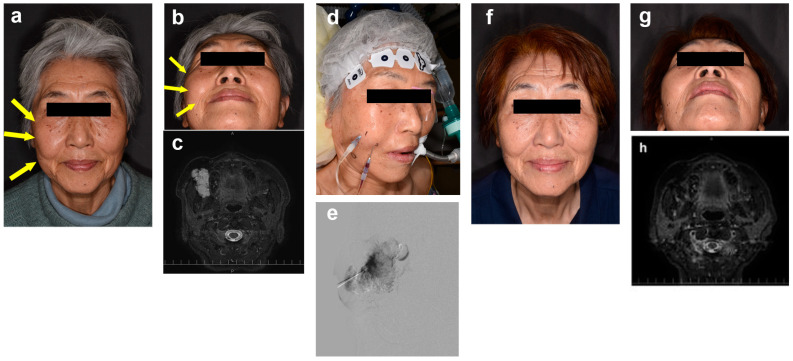
A 78-year-old female with a painful intramuscular venous malformation (VM) in the right buccal region. ((**a**,**b**), yellow arrows) Pre-sclerotherapy photographs (frontal and low-angle views). The yellow arrows indicate the lesion. (**c**) Pretreatment STIR MRI showing the lesion volume measured independently by two radiologists as 20.5 cm^3^ and 19.4 cm^3^ (mean: 20.0 cm^3^). (**d**,**e**) Photographs during sclerotherapy. Following the established protocol, 8.2 mL of 5% ethanolamine oleate (EO) was injected percutaneously under digital subtraction angiography (DSA) via direct percutaneous puncture. (**f**,**g**) Photographs 3 months after sclerotherapy (frontal and elevated-angle views). (**h**) Post-treatment STIR MRI showing lesion volumes measured as 6.4 cm^3^ and 6.3 cm^3^ (mean: 6.4 cm^3^), corresponding to a 68.2% reduction.

Sclerotherapy involved direct lesion puncture under ultrasound guidance, with blood backflow confirmed via negative pressure to ensure proper needle placement. Digital subtraction angiography (DSA) was employed to visualize the lesion following the injection of contrast medium, after which 5% EO diluted 1:1 with contrast or saline was administered. The dosage was capped at 0.4 mL/kg, with a maximum volume of 30 mL per session (Figure 1). The administration protocol adhered to safety guidelines established for the treatment of gastric varices in Japan [11], with efficacy in VM management supported by prior studies [7,12,13]. A urethral catheter was inserted at the discretion of the principal investigator. Urine color was monitored hourly for several hours following sclerotherapy. In cases of hemoglobinuria, 2000–4000 units of haptoglobin were administered intravenously without delay. Routine laboratory evaluations, including complete blood count, serum biochemistry, basic metabolic panel, and coagulation profiles, were performed preoperatively and on postoperative day 1 (POD1).

### 2.4. Outcomes

The primary exploratory outcome was the comparison of coagulation factor levels before and after sclerotherapy. Secondary outcomes included the evaluation of associations between patient demographics, lesion characteristics, and post-sclerotherapy details, as well as the therapeutic effect of sclerotherapy on VM. Additionally, the relationship between lesion type, the lesion reduction effect of sclerotherapy, and coagulation factors was evaluated.

### 2.5. Statistical Analysis

Data analyses were performed using BellCurve for Excel (version 4.07; Social Survey Research Information Co., Ltd., Tokyo, Japan). Continuous variables were expressed as mean ± standard deviation. Fisher’s exact test was used to compare categorical variables between the achieved and non-achieved groups. Statistical tests, including the Wilcoxon matched-pairs signed-rank test, Mann–Whitney U, and Spearman’s rank correlation coefficient, were applied as appropriate. A *p*-value < 0.05 was considered statistically significant. Given that this is a hypothesis-exploratory study with post-hoc analysis, multiplicity of tests was not considered. Since nonparametric tests were used, Cliff’s delta was calculated instead of posterior power. When required, 95% confidence intervals (CIs) for the estimates were calculated.

## 3. Results

### 3.1. Patient Demographics and Lesion Characteristics

Between January 2021 and April 2023, 44 patients underwent sclerotherapy with EO for VMs. These patients were included in this retrospective post-hoc analysis. Patient demographics are summarized in Table 1.

The reliability of the 88 lesion volume measurements taken before and after treatment by two independent radiologists was high, with an ICC of 0.999 (95% CI: 0.998–1.000). Target lesion sizes were significantly smaller for cystic-type VMs compared to diffuse-type VMs (Wilcoxon matched-pairs signed-rank test, *p* = 0.017). Coagulation parameters, including D-dimer and fibrinogen levels, were not significantly different between cystic and diffuse lesions. Localized intravascular coagulopathy (LIC), defined as D-dimer >1.0 µg/mL and fibrinogen <180 mg/dL, was observed in four patients, each of whom underwent a single sclerotherapy session. The median EO dose was 9.00 mL (range: 2.00–22.1 mL), with no significant difference in dose per lesion volume or per body weight between cystic (median: 8.35 mL) and diffuse (median: 12.2 mL) lesion types (Table 2).

A ≥ 20% reduction in lesion volume was achieved in 26 of 44 patients (59.1%). Cystic lesions had higher success rates (72.7%, 95% CI: 51.85–86.85%) compared to diffuse lesions (45.5%, 95% CI: 26.92–65.34%) (Table 3).

### 3.2. Coagulation Parameter Changes

**Figure 2:**
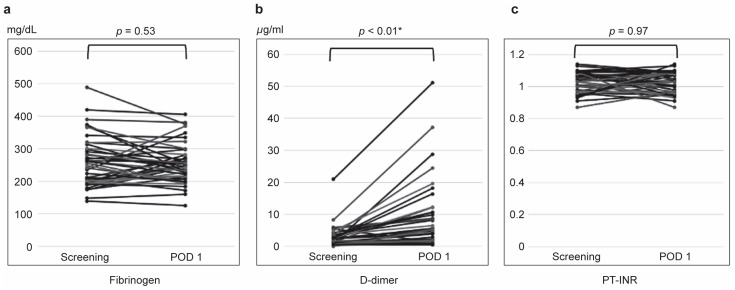
Trends in coagulation markers: fibrinogen (**a**), D-dimer (**b**), and PT-INR (**c**). Screening = pretreatment (within 30 days prior to the procedure), POD1 = within 24 h after the procedure. The black line represents cystic-type VMs, and the gray line represents diffuse-type VMs. D-dimer levels significantly increased post-treatment compared to baseline. Fibrinogen and PT-INR levels showed no significant changes between screening and POD1. * indicates statistically significant.

On POD1, D-dimer levels significantly increased compared to baseline (*p <* 0.001). Fibrinogen levels showed a non-significant decrease (*p* = 0.53), and PT-INR values remained unchanged (*p* = 0.97) (Figure 2).

### 3.3. Achieved vs. Non-Achieved Groups

**Figure 3:**
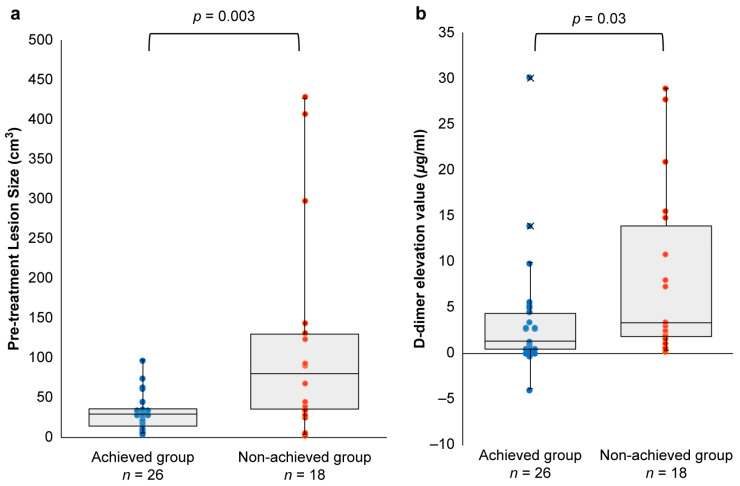
Comparison of characteristics between the achieved and non-achieved groups. (**a**) Pretreatment lesion sizes for all 26 cases in the achieved group were <100 cm^3^. The Mann–Whitney U test revealed a significant difference in pretreatment lesion size between the achieved and non-achieved groups (Cliff’s Delta = −0.54, 95%CI: −0.78–0.16. *p* = 0.003). (**b**) D-dimer elevation was significantly higher in the non-achieved group compared to the achieved group, as determined by the Mann–Whitney U test (Cliff’s Delta = −0.38, 95%CI: −0.65–0.027, *p* = 0.03).

Patient and lesion characteristics between the achieved group (*n* = 26) and non-achieved (*n* = 18) groups are summarized in Table 4. Among 38 patients with pretreatment volumes < 100 cm^3^, 68.4% (26/38) were in the achieved group. Lesion type (cystic vs. diffuse) did not significantly influence lesion reduction achievement (*p* = 0.12). Pretreatment lesion sizes were significantly smaller in the achieved group (*p* = 0.003) (Figure 3a).

Regarding the use of 5% EO, there was no significant difference between the two groups in terms of total usage, amount used per lesion, or amount used per body weight. In the achieved group, a significant positive correlation was observed between the volume reduction rate at POD1 and the amount of 5% EO administered per lesion volume (mL/cm^3^) (Spearman’s ρ = 0.43, 95% CI: 0.072–0.71, *p* = 0.03).

Compared to baseline, D-dimer levels on POD1 were significantly higher in the non-achieved group than in the achieved group (*p* = 0.03) (Figure 3b). However, within the achieved group, no significant correlation was found between the rate of volume reduction at 3 months post-treatment and the change in D-dimer levels from baseline to POD1 (Spearman’s ρ = −0.32, 95% CI: −0.63–0.081, *p* = 0.12).

### 3.4. Safety Evaluations

No death or serious adverse events requiring hospitalization were reported. Common complications, such as skin necrosis and visual impairment, which were of particular concern, were not observed. Adverse events were recorded in 42 patients (95.5%). Of these, there was 1 case of a grade 3 adverse event, 9 cases of grade 2 events, and 32 cases of grade 1 events. The single grade 3 adverse event was related to pain. Grade 2 adverse events included pain in three cases, hemoglobinuria in two cases, and individual cases of swelling, increased D-dimer levels, increased serum creatine phosphokinase levels, and transient ulnar nerve paralysis. All hemoglobinuria cases occurred on the day of sclerotherapy and were managed with intravenous haptoglobin (2000–4000 U). All patients recovered without any sequelae.

## 4. Discussion

Sclerotherapy is widely employed for treating VMs of the body surface, with numerous studies corroborating its efficacy and safety [2,6,7,8,9,14]. While changes in coagulation markers post-sclerotherapy are acknowledged, their detailed dynamics remain insufficiently explored. To our knowledge, this is the first study to evaluate the impact of EO sclerotherapy on coagulation markers in conjunction with precise volumetric VM measurements.

Previous studies, such as Vollherbst et al., have utilized precise volumetric measurements by manually delineating VM lesions on each imaging slice, predominantly using the axial plane but incorporating sagittal or coronal planes when needed [15]. Similarly, we conducted manual segmentation, ensuring accuracy through analysis by two independent radiologists, thus maintaining high measurement quality.

The selection of 5% EO in this study stems from its widespread use in Japan for sclerotherapy of esophageal and gastric varices, as well as VMs, with well-documented safety and efficacy. Among sclerosing agents, EO demonstrates a safer profile than ethanol [12] while offering greater therapeutic efficacy than polidocanol [16]. Sodium tetradecyl sulfate (STS), another commonly used agent, is not available in Japan, positioning EO as the preferred agent for VM sclerotherapy due to its balance between therapeutic efficacy and safety.

In terms of treatment efficacy, 26 of 44 patients achieved a VM volume reduction of ≥20% 3 months post-sclerotherapy. Specifically, 16 patients (72.7%) with cystic lesions and 10 patients (45.5%) with diffuse lesions experienced a volume reduction of over 20%, consistent with prior findings [7]. Furthermore, a systematic review by Horbach et al. reported an overall response range of 88–100% for sclerotherapy using EO in 188 cases of VM [17]. Smaller lesions (<100 cm^3^) demonstrated significantly greater reductions compared to larger lesions (≥100 cm^3^), highlighting the higher efficacy of EO in reducing smaller VM volumes. No significant differences were observed between achieved and non-achieved groups concerning the total dose, dose per lesion, or dose per body weight of EO. These results suggest that irrespective of the EO dosage, smaller lesions are more likely to achieve volume reduction, while larger, diffuse lesions are less likely to respond sufficiently in terms of volume reduction. Notably, in the achieved group, the volume reduction rate correlated significantly with the EO volume per lesion, suggesting that pretreatment volumetry could guide the optimal EO dose. On average, a dose of 0.42 mL/cm^3^ per lesion appears ideal.

Regarding coagulation markers, Leung et al. measured D-dimer levels after alcohol sclerotherapy in 18 pediatric patients with VMs. They observed increased D-dimer levels in 12 patients post-treatment, with satisfactory clinical outcomes after 6 months in 11 cases and no long-term recurrence in 10 cases [18]. They concluded that elevated D-dimer levels after alcohol-based sclerotherapy might predict early treatment response and reduce long-term re-expansion rates. Similarly, Mason assessed coagulation markers pre- and post-sclerotherapy using dehydrated alcohol or STS for VMs, observing significant increases in D-dimer levels, decreases in platelets and fibrinogen, and prolonged prothrombin time immediately post-treatment. The decrease in fibrinogen was transient, returning to baseline within 24 h, but changes in platelets, D-dimer, and prothrombin time persisted [19]. Likewise, Yu et al. evaluated coagulation profiles and clinical outcomes in 61 patients treated with absolute alcohol sclerotherapy. They reported increases in fibrin degradation products (FDP) and D-dimer levels, which correlated positively with treatment outcomes [20].

In our study, D-dimer levels significantly increased on POD1, while fibrinogen showed a trend toward a decrease but did not differ significantly. Importantly, fibrinogen levels showed a non-significant downward trend but remained above 100 mg/dL in all cases. The change in D-dimer levels from baseline to POD1 was significantly higher in the non-achieved group than in the achieved group. Contrary to our initial assumptions, greater D-dimer changes were associated with poorer volume-reduction outcomes. EO induces sclerosis via endothelial damage, leading to fibrin-product deposition and thrombosis within hours of exposure. Its superior thrombogenic effect further enhances the efficacy of EO sclerotherapy. Additionally, the oleate component triggers an inflammatory response that extends beyond the vessels into the surrounding tissues [21]. In VM sclerotherapy, therapeutic effects are achieved when the sclerosing agent remains within the lesion, causing vascular endothelial damage, thrombus formation, and subsequent inflammation. Therefore, efficient anchorage of the sclerosing agent within the lesion enhances therapeutic outcomes. In the achieved group, the sclerosing agent remained efficient within the lesion, whereas in the non-achieved group, it did not remain as effective. This suggests that the thrombus formed after EO injection flowed into the systemic circulation in the non-achieved group, resulting in significant changes in D-dimer levels. Furthermore, previous studies have not examined the correlation between accurate MRI volumetric measurements and coagulation factors, which may explain the differences observed in our results.

To our knowledge, this study is the first to correlate MRI-based volumetric measurements with coagulation markers and treatment outcomes. Our findings suggest that lesion size, morphology, and drainage characteristics influence therapeutic efficacy, providing insights into predicting treatment outcomes and selecting suitable candidates for EO sclerotherapy.

In terms of safety, hemoglobinuria was observed in 23 patients (52.2%), consistent with previous reports (27.7–50.0%) [7,12,22]. Hemoglobinuria following sclerotherapy with EO poses a potential risk of acute renal failure; therefore, clinicians are encouraged to be vigilant for timely intervention [23]. In this study, prompt administration of intravenous haptoglobin effectively resolved symptoms in all cases without progression to renal failure. This highlights the importance of vigilant monitoring and timely intervention during EO sclerotherapy.

This study has some limitations. First, it represents a retrospective post-hoc analysis of a prospective, single-arm trial with a relatively small sample size. Variability in coagulation markers and the inclusion of four cases with localized intravascular coagulation may have affected the findings. Second, post-intervention MRI assessments were limited to 3 months; extended follow-up is needed to validate our findings. The significantly smaller baseline volume in cystic lesions compared to diffuse lesions may have influenced the quality of the group comparisons. Third, while MRI-based morphological classifications ensured quality, distinctions between cystic and diffuse VMs were subjective and not supplemented by angiographic evaluation.

Given the relatively small sample size, it is difficult to generalize the results. Future controlled prospective studies will be essential; however, the findings in this study may provide valuable insights for clinicians.

## 5. Conclusions

This study is the first to evaluate the therapeutic efficacy of PS with EO for VMs using precise volumetric MRI measurements in a multicenter clinical trial. Additionally, it explores the association between early post-treatment coagulation markers and treatment outcomes. Notably, a greater increase in D-dimer levels from screening to POD1 was observed in patients with <20% volume reduction, indicating that D-dimer elevation does not positively correlate with treatment response.

## Figures and Tables

**Table 1:** Demographic characteristics of patients undergoing percutaneous sclerotherapy.

			All (*n* = 44)	Venous Malformations		*p*-Value
				Cystic-Type	Diffuse-Type	
				(*n* = 22)	(*n* = 22)	
Sex	Male, *n* (%)			13 (59.1)	9 (40.9)	0.37
	Female, *n* (%)			9 (40.9)	13 (59.1)	
Age, median, year (range)				16.5 (3−78)	16.5 (5−59)	0.91
Weight, median, kg (range)				49.1 (14.7−92.0)	50.5 (20.3−75.2)	0.24
Lesion, *n* (%)	Trunk		9	5 (22.7)	4 (18.2)	
	Extremity	Upper	11	3 (13.6)	8 (36.4)	
		Lower	15	5 (22.7)	10 (45.5)	
	Head and neck	Head	0	0 (0.0)	0 (0.0)	
		Face (except for intraoral)	5	5 (22.7)	0 (0.0)	
		Intraoral	1	1 (4.5)	0 (0.0)	
		Neck	3	3 (13.6)	0 (0.0)	
Size, median (min, max) cm^3^			28.1 (2.25, 429)	27.9 (2.25, 131)	62.1 (3.85, 429)	0.017 *
Coagulation markers	Fibrinogen, median (min, max) mg/dL		246 (139, 489)	211 (139, 489)	262 (175, 374)	0.39
	D-dimer, median (min, max) μg/mL		1.40 (0, 21.0)	2.05 (0.5, 21.0)	1.10 (0, 8.30)	0.35
	PT-INR, median (min, max)		1.03 (0, 1.14)	1.03 (0.91, 1.13)	1.03 (0.87, 1.14)	0.98
Previous treatment for VM, *n* (%)	Sclerotherapy		22 (50.0)	10 (45.5)	12 (54.5)	
	Surgical resection		6 (13.6)	4 (18.2)	2 (9.1)	

VM, venous malformation. In evaluations of age, weight size, and coagulation markers, the values of *p* were based on nonparametric Wilcoxon’s signed-rank test. * indicates statistically significant.

**Table 2:** Treatment details for patients receiving monoethanolamine oleate (EO).

		All	Cystic-Type	Diffuse-Type	*p*-Value
		(*n* = 44)	(*n* = 22)	(*n* = 22)	
5% EO	Total volume, median (min, max) mL	9.00 (2.00, 22.1)	8.35 (2.00, 22.0)	12.2 (2.60, 22.1)	0.17
	Amount used per lesion, median (min, max) mL/cm^3^	0.26 (0.04, 0.40)	0.25 (0.04, 0.40)	0.31 (0.05, 0.40)	0.15
	Amount used per body weight, median (min, max) mL/kg	0.27 (0.04, 1.78)	0.31 (0.10, 1.16)	0.27 (0.04, 1.78)	0.27

The values of *p* were based on nonparametric Wilcoxon’s signed-rank test.

**Table 3:** Characteristics of patients achieving ≥20% venous malformation volume reduction at 3-month follow-up.

	No. of Patients	Achieving a Reduction of at Least 20%		
		No. of Achievers	Percentage	95% Confidence Intervals
All	44	26	59.1	44.41–72.31
Cystic type	22	16	72.7	51.85–86.85
Diffuse type	22	10	45.5	26.92–65.34

**Table 4:** Comparative analysis of achieved (≥20% volume reduction) and non-achieved groups post-sclerotherapy.

		All (*n* = 44)	Venous Malformations		*p*-Value
			Achieved Group	Non-Achieved Group	
			(*n* = 26)	(*n* = 18)	
Type of lesion, *n* (%)					0.12
	Cystic type	22	16 (72.7)	6 (27.3)	
	Diffuse type	22	10 (45.5)	12 (54.5)	
Pre-treatment lesion size, median (IQR) cm^3^			27.9 (12.6, 35.0)	79.2 (34.3, 129.3)	0.003 *
5% EO	Total volume, mean (±SD)		8.9 (±5.0 mL)	11.5 (±5.5 mL)	0.10
	Amount used per lesion, mean (±SD)		0.42 (±0.29 mL/cm^3^)	0.37 (±0.43 mL/cm^3^)	0.40
	Amount used per weight, mean (±SD)		0.27 (±0.22 mL/kg)	0.31 (±0.10 mL/kg)	0.31
D-dimer elevation value, median (IQR) μg/mL †			1.20 (0.33, 4.2)	3.2 (1.7, 13.8)	0.03 *

† indicates increase value of D-dimer in postoperative day 1 from screening. * indicates statistically significant.

## Data Availability

Individual participant data underlying the results (text, tables, and figures) reported in this article after de-identification will be shared following article publication. Requests will be honored by researchers who provide a methodologically sound proposal and execute a data-use agreement with Kyorin University. Requests should be directed to the corresponding author by email.
